# Selective Loss of Cysteine Residues and Disulphide Bonds in a Potato Proteinase Inhibitor II Family

**DOI:** 10.1371/journal.pone.0018615

**Published:** 2011-04-11

**Authors:** Xiu-Qing Li, Tieling Zhang, Danielle Donnelly

**Affiliations:** 1 Potato Research Centre, Agriculture and Agri-Food Canada, Fredericton, Canada; 2 Department of Plant Science, McGill University, Ste-Anne-de-Bellevue, Canada; University of Wyoming, United States of America

## Abstract

Disulphide bonds between cysteine residues in proteins play a key role in protein folding, stability, and function. Loss of a disulphide bond is often associated with functional differentiation of the protein. The evolution of disulphide bonds is still actively debated; analysis of naturally occurring variants can promote understanding of the protein evolutionary process. One of the disulphide bond-containing protein families is the potato proteinase inhibitor II (PI-II, or Pin2, for short) superfamily, which is found in most solanaceous plants and participates in plant development, stress response, and defence. Each PI-II domain contains eight cysteine residues (8C), and two similar PI-II domains form a functional protein that has eight disulphide bonds and two non-identical reaction centres. It is still unclear which patterns and processes affect cysteine residue loss in PI-II. Through cDNA sequencing and data mining, we found six natural variants missing cysteine residues involved in one or two disulphide bonds at the first reaction centre. We named these variants Pi7C and Pi6C for the proteins missing one or two pairs of cysteine residues, respectively. This PI-II-7C/6C family was found exclusively in potato. The missing cysteine residues were in bonding pairs but distant from one another at the nucleotide/protein sequence level. The non-synonymous/synonymous substitution (Ka/Ks) ratio analysis suggested a positive evolutionary gene selection for *Pi6C* and various *Pi7C*. The selective deletion of the first reaction centre cysteine residues that are structure-level-paired but sequence-level-distant in PI-II illustrates the flexibility of PI-II domains and suggests the functionality of their transient gene versions during evolution.

## Introduction

Many enzymes and proteins such as members of the potato proteinase inhibitor II superfamily have disulphide bonds (disulphide bridge, SS-bond) formed between the thiol groups of cysteine residues. Although the amino acid residue methionine also contains sulphur, methionine cannot form disulphide bonds. The disulphide bond, usually formed between different regions or peptides, is relatively strong. Its typical bond dissociation energy is 60 kcal/mole. This is approximately 71% of the strength of a typical peptide backbone carbon-carbon bond (dissociation energy of 83–85 kcal/mole). Disulphides perform diverse functions in proteins, from maintaining the folding and stability of proteins to preserving bioactive structure essential to specific protein function [1], [2], [3], [4], [5].

Analysis of naturally occurring variants can reveal insights into the natural selection and evolution of disulphide bond-containing proteins. Disulphide bonds were thought to be generally very well conserved in proteins [6]. However, a recent large scale analysis on structural features in homologous protein domain families of known 3-D structures reported that only 54% of disulphide bonds compared between homologous pairs are conserved [7]. The same study also found that the elimination of a disulphide in a homologue need not always result in more stable interactions between equivalent residues, and about 35% of the poorly conserved disulphides show gaps in their alignment [7]. The non-conserved disulphides have variable structural features that were thought to be associated with differentiation or specialisation of protein function [7].

In globular proteins, there is a strong preference for relatively shorter connections; the average separation for cysteine residues within a disulphide bond is 15 residues [6]. The loss of a disulphide bond in a globular protein is sometimes from losing both [6], or only one, of the two cysteine residues [8]. In an antithrombin deficiency family, the disruption of a disulphide bond due to the loss of a cysteine residue left a free cysteine residue and an unconstrained C-terminus [8].

After the loss of a cysteine residue, a new pairing can sometimes occur between the remaining cysteine residues in the protein. For example, a mutated anti-Mullerian hormone type II (AMHRII) receptor gene encoding a protein lacking one of the cysteine residues leads to persistent Mullerian duct syndrome in human males [9]. In the wild type protein, the C5 cysteine residue forms a bridge with the C8 cysteine residue. However, AMHRII contains no C8 cysteine residue. Instead, its C5 cysteine residue is predicted to form a disulphide bridge with a C (non-exist in other similar proteins analyzed) that is directly adjacent to C3 [9].

Behe and Snoke [10] proposed models for simulating evolution of protein features that require multiple amino acid residues such as the case of disulphide bonds using a conceptually simplest route-point mutation system in duplicated genes. These authors consider a situation in which the intermediate steps to a new protein are neutral and involve non-functional products. This view was challenged by Lynch [11] who proposed a neofunctionalization model assuming that the intermediate step towards a two-residue adaptation is non-debilitating with respect to the original function and effectively neutral. Clearly, protein evolution involving disulphide bonds is still actively debated, and illustrations of natural variants can promote understanding of natural selection and evolutionary process(es) of genes encoding disulphide bond-containing proteins.

One of the disulphide bond-containing protein families is the potato proteinase inhibitor type II (PI-II, or Pin2, for short) superfamily, which is found in most solanaceous plants and participates in plant development, wound response, and defence [12]. Each PI-II domain, or repeat at the primary sequence level, contains eight cysteine residues (8C), and two domains (usually non-identical) forming a functional proteinase inhibitor II protein with eight disulphide bonds [13], [14]. The sequence of the PI-II repeats is quite variable; only the eight cysteine residues involved in the disulphide bonds and a single proline residue are strictly conserved in each domain in different type II proteinase inhibitors identified in solanaceous species (http://www.sanger.ac.uk//cgi-bin/Pfam/getacc?PF02428). The proper folding is important to the proteinase inhibition activity [15]. Each eight-cysteine-residue sequence region was usually termed a domain, but amino acid sequences of the domain are different. The functional protein needs two such non-identical domains to fold together to form the eight disulphide bonds and the two reaction centres. PI-II belongs to one of ten recognized types of plant proteinase inhibitors [16].

The PI-II protein has a double-head-like structure with one reaction centre at each head [13], [17]. Each head mainly consists of five amino acids in an array at the primary sequence level [15], [18]. This array has two conserved cysteine residues with three amino acid residues between them. These two conserved cysteine residues pair with the two counterpart cysteine residues in another domain to form two disulphide bonds. However, the three internal amino acid residues can be modified through genetic engineering. In *Nicotina alata*, artificial deletion of two disulphide bonds reveals that one of the bonds is essential for protein binding to trypsin while the other bond markedly decreases the timescale of motion [19].

The primary sequence-level domains of the PI-II peptide interact to form double-headed proteins although the two primary domains can be encoded from two discontinuous parts of the gene [13], [15], [17], [20]. This is different from the relatively well studied globular proteins [6]. In PI-II, there are several unknown features, including the pattern by which disulphide bond partners may lose cysteine residues at the reaction centres, and whether this loss was random or selective. Furthermore, it is not known whether the intermediate versions were functional during this evolutionary process.

The two cysteine residues of the five amino acid residue array at each of the two reaction centres are apparently essential as they are always present in reported natural variants [15]. The three amino acid residues between the two cysteine residues often differ between homologs; this is important for both function and specificity [15]. The amino acid sequence of the first reaction centre in potato PI-II is “CTLEC”, and the second reaction centre is “CPRNC”. Transcript sequence information for solanaceous plants has increased rapidly due to new developments in DNA sequencing. This increases the possibility of finding new variants of PI-II.

We have previously reported the constitutive expression of a PI-II superfamily gene (cDNA C463) cloned from potatoes [21]. Now, in the current study, we found that one of eight cysteine residue pairs is missing in the predicted protein encoded by C463, and further bioinformatic analysis of all available potato gene sequences led to the identification of PI-II potato genes with one or two cysteine residue pairs missing in their encoded proteins. We named these genes *Pi7C* and *Pi6C*, and their encoded proteins Pi7C and Pi6C (for the missing one or two pairs of cysteine residues, respectively). Here, we report on these *Pi7C* and *Pi6C* genes, describe the PI-II domain flexibility, and investigate the evolutionary selection process that lead to the emergence of these two genes.

## Results

### cDNA cloning and nucleotide sequence analysis of *Pi7C*

The DNA sequence of the cDNA clone C463 (*Pi7Ca*, GenBank accession EF469204) from the diploid potato clone (DC) 11379-03 showed 88% identity (BLASTn Expect = 3e-38) in March 2007 to the tomato auxin-induced proteinase inhibitor type II gene (*ARPI* TR8, gi|408007 for genomic DNA, and gi|405581 for mRNA) [22], [23]. However, this C463 DNA sequence did not show significant similarity to any known potato genes in BLASTn and BLASTX searches in March 2007, not even to the known PI-II gene of potato [20]. Only one potato mRNA (gb|EU368949.1) was found in BLASTn GenBank Nucleotide Collection (nr/nt) on December 24, 2010 (99% identity, Expect = 0.0). A BLASTp search using the known potato PI-II (ABR29625) detected C463 *Pi7Ca* as a distant (Identities = 66/159, 42%) but significantly similar (Expect = 2e-23) protein on December 24, 2010.

The cDNA clone C463 contained a full length open reading frame. The mRNA-encoded peptide was 152 amino acids long. Both its starting (NH_2_-terminal) and ending (COOH-terminal) regions were similar to the *ARPI* protein, but the internal region was quite different from ARPI with an identity of 92% (24 aa), 66% (101 aa), and 93% (27 aa) for the leader, middle, and tail regions, respectively. In PSI- and PHI-BLAST analysis, the amino acid sequence (152 aa) showed two proteinase inhibitor-II domains at the primary sequence level with similarity to the tomato ARPI peptide (Q43710.1, Identities = 107/154 (70%), Expect = 9e-45, on December 24, 2010) and PI-II from several *Nicotiana* species with the greatest to *N. cavicola* PI-II gb|ABA42891.1 (56% identity, Expect = 5e-4, on December 24, 2010). The first 24 or 22 amino acids of the *Pi7Ca*-encoded product were nearly identical to the signal peptide of ARPI targeting signal peptide with differences only in three amino acids. The similarity analysis on both the DNA sequence and the decoded-protein sequence suggested that the C463 clone is in the family of a type II proteinase inhibitor. For this reason, the cDNA C463's identified gene from the DC was called *Pi7Ca*.

**Figure 1:**
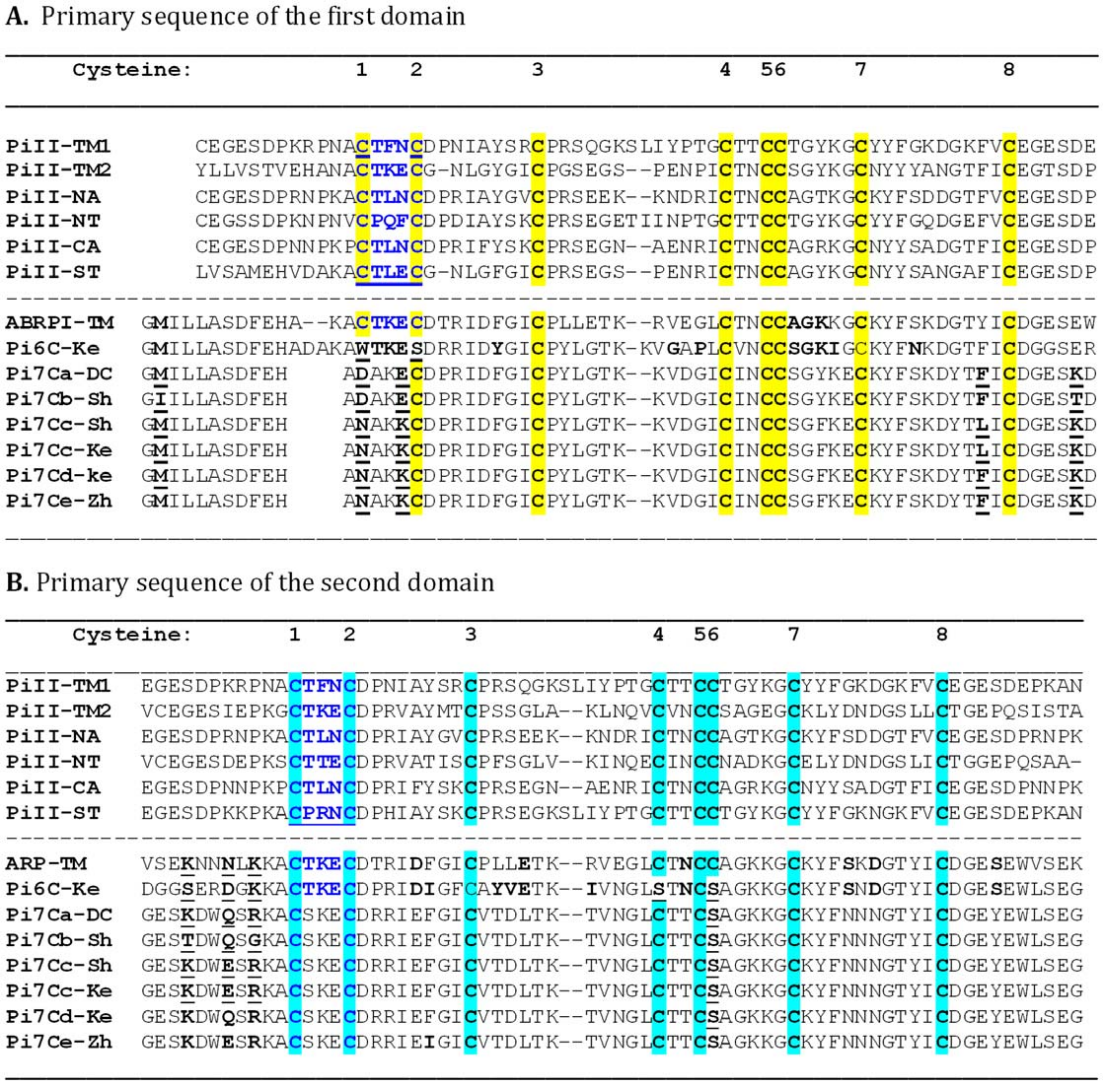
Amino acid sequence alignment of the conserved PI-II-like domains between different species. **PiII:** Potato Proteinase Inhibitor II; **TM:** tomato (*Solanum lycopersicum* (formerly *Lycopersicon esculentum*); **NA:** *Nicotiana alata*; **NT:** tobacco (*N. tabacum*); **CA:** pepper (*Capsicum annuum*); **ST:** potato (*S. tuberosum*) cultivar Bintje; **Ke:** from potato (*S. tuberosum*) cultivar Kennebec. **DC:** the diploid line 11379-03, a mainly *S. tuberosum*- and *S. phureja* and *S. stenotomum*-derived material. **Sh:** potato (*S. tuberosum*) cultivar Shepody; **Zh:** *S. tuberosum* cultivar Zhongshu No. 3. The protein names and GIs are described in File S1. The reaction centre CTLEC and CPRNC of standard 8C PI-II (PiII-ST) in potato were written in dark blue. The conserved cysteine residues were highlighted either in yellow for the primary-sequence-level Domain 1 or in light greenish blue for the primary sequence-level Domain 2. The bold or bold-underlined amino acids are the ones showing the polymorphisms discussed in the text. Note that the 1^st^ C and 2^nd^ C in the first domain and the 4^th^ C and 6^th^ C in the second domain are missing in some genes and that the 7C-domain regions are more conserved than the upstream and the inter-domain regions among *Pi7C* alleles.

BLAST search against the non-human EST databases in GenBank identified 62 highly similar ESTs of potato, mainly from *S. tuberosum* ‘Shepody’ [24], ‘Kennebec’, and ‘Bintje’ (http://jcvi.org/potato/) in March 2007. Four different sequences (*Pi7Ca*, *Pi7Cb*, *Pi7Cc*, and *Pi7Cd*) of *Pi7C*, each encoding a 152-amino-acid peptide, were identified. An updated BLASTp search added another member (gi|165906348) (named, *Pi7Ce*) on December 24, 2010. *Pi7Ca* was from the DC, *Pi7Cb* and *Pi7Cc* from ‘Shepody’, *Pi7Cd* from ‘Kennebec’, and *Pi7Ce* from ‘Zhongshu No. 3’. They all encoded two domains. The tomato PI-II (ARPI) has three PI-II domains. Therefore, all the potato Pi7C proteins are different from the tomato PI-II in having one fewer domain. The amino acid sequence alignment showing the cysteine residue deletion on the two domains is shown in Figure 1.

### Identification and feature analysis of Pi6C

When *Pi7Cd* (gi|13613799) was used in a BLAST search against the “est_others” database in GenBank, 10 similar ESTs (such as gi|12587033, gi|21916076) could be decoded into a peptide of 156 amino acids. The peptide carried two putative PI-II domains with six cysteine residues (6C) per domain (Figure 1). The 1^st^ and 2^nd^ cysteine residues of the first domain and the 4^th^ and 6^th^ cysteine residues of the second domain were absent (Figure 1).

### Novel feature of *Pi7C* at the primary sequence level

Comparison of the conserved primary sequences between Pi7C and other PI-II domains allowed the discovery that each of the two Pi7C domains had only seven cysteine residues (7C), instead of the standard eight cysteine residues (8C). Since each PI-II functional protein is formed by two non-identical domains, the Pi7C protein is expected to miss one cysteine residue per domain and two cysteine residues (i.e., one pair) for the protein, compared with the conserved Pi8C protein. The two non-identical Pi7C domains are on the same peptide. This *Pi7C* encoded peptide did not have the first cysteine residue of the first domain, nor the sixth cysteine residue of the second domain. As underlined in Figure 1, the first cysteine residue (letter C) of Pi7Ca-DC and Pi7Cb-Sh was replaced by aspartate (letter D). The sixth C in the second domain was replaced by serine (letter S). The absence of two cysteine residues with their corresponding disulphide bonds is expected to cause protein structural change.

### Domain numbers in Pi6C, Pi7C, and related PI-II peptides

Many PI-II peptides have multiple domains. For example, *N. sylvestris* PI-II (gi|76446054) has six PI-II domains, tomato PI-II *ARPI* (gi|405581) has three, but Pi6C and Pi7C have only two domains. The standard PI-II peptide (protein gi|AAA53278) of potato also has two PI-II domains. None of the potato ESTs picked up in the BLASTn search of March 2007 showed a different domain number. Of course, this result does not rule out the possibility of domain number variants within species because we did not analyze all paralogs of PI-II genes in all species. Further research is required to verify whether reduction in domain number is an evolutionary tendency from the ancestral species to the current tetraploid agricultural potato cultivars.

### Conservation among Pi7C members

The availability of different Pi7C members permits analysis of which regions are more conserved than others along the Pi7C peptide. The amino acid length (152 amino acids) and the 7C were well conserved between the members, with polymorphism mainly upstream of the first domain and the inter-domain region. The leader-like first 13 amino acids (data not shown), the core domain regions, and the end area after the second domain, were 100% conserved among the Pi7C members analyzed. The results suggest the following: 1) The first and second domains may interact closely to work together for the gene's function; 2) The leader sequence and the region after the second domain are important functionally and were therefore conserved; 3) The length of the outside of the proteinase inhibitor domain region is also important, suggesting that the sequence outside the domain region also contributes to maintaining the structure of the *Pi7C* protein. The amino acid sequences, except the 8 C's, are not highly conserved between different 8C PI-II genes (Figure 1).

### Estimated structural level change in *Pi6C* and *Pi7C*

**Figure 2:**
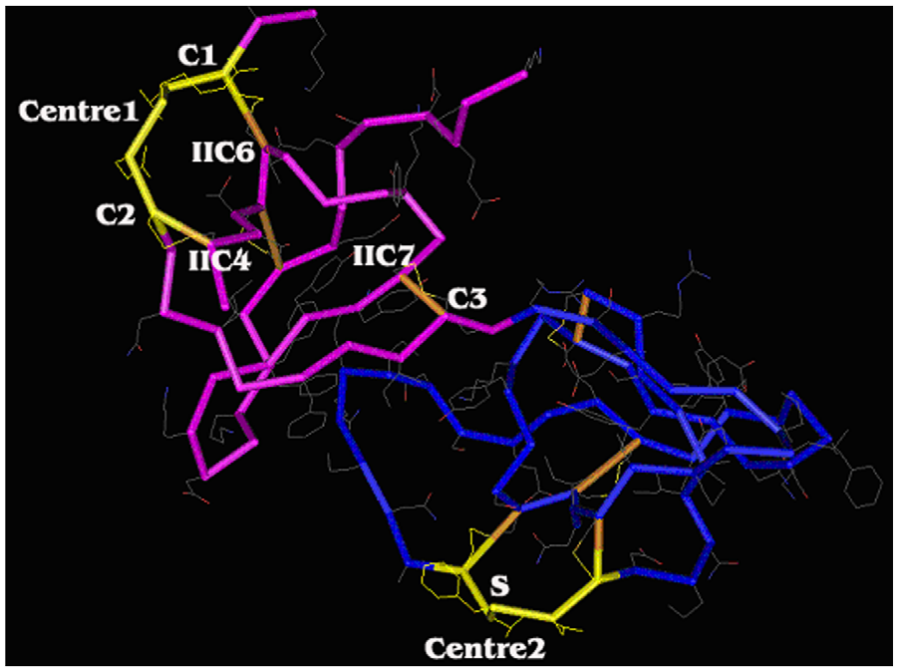
Indication of the cysteine-residue-missing locations of *Pi7C* protein on the standard structure of potato type II proteinase inhibitor (PI-II, pfam02428.12). The graph is turned in a way that the missing cysteine residues can be labelled. The double-headed structure formed jointly by two domains. **C1, C2, C3:** The 1^st^, 2^nd^, and 3^rd^ cysteine residues in the first domain (at the primary-sequence level). **IIC4, IIC6, IIC7:** the 4^th^, 6^th^, and 7^th^ cysteine residues in the second domain (at the primary-sequence level). **Centre 1:** The 1^st^ reaction site (5 a.a., from C1 to C2, inclusive; marked in light yellow). **Centre 2:** The second reaction site (5 a.a. from IIC1 to IIC2, inclusive; marked in light yellow). **S:** The T (threonine) on the 2^nd^ reaction centre was replaced by S (serine) in the 7C domain members (*Pi7Ca*, *Pi7Cb*, *Pi7Cc*, *Pi7Cd*, and *Pi7Ce*). The brown color indicates the disulphide bonds formed between paired cysteine residues. Note that without C1-IIC2 bond and C2-IIC4 bond, the 1^st^ reaction site no longer exists and the structure of the protein will be considerably changed.

Pi6C and Pi7C are likely different in structure from the standard PI-II due to the deletion of disulphide bond(s). The location of cysteine residue changes in *Pi6C* and *Pi7C* are illustrated on the standard PI-II domain at the protein structural level (pfam02428.12) (Figure 2). Both *Pi6C* and *Pi7C* lack the 1^st^ cysteine residue (C1) on the first domain and the 6^th^ cysteine residue (IIC6) on the second domain. These two cysteine residues in PI-II peptides pair to form a disulphide bond. In Pi6C and Pi7C, this bond would not exist. In *Pi6C*, the 2^nd^ cysteine residue (C2) of the first domain and the 4^th^ cysteine residue (IIC4) on the second domain were also absent. These two cysteine residues are paired in PI-II. Their absence in *Pi6C* should cause the absence of this disulphide bond. In *Pi6C*, the absence of both the C1-IIC6 and the C2-IIC4 bonds may cause the long chain from the N-terminal up to the C3 location to change its folding. The structure of the left head (pink coloured part in Figure 2), normally in the standard PI-II, would largely be destroyed/changed in Pi6C.

The reaction centres were also substantially changed in Pi6C and Pi7C. The first PI-II reaction centre would largely lose activity due to the absence of the one (C1) or both pairs of cysteine residues (C1, C2) of the centre (Figure 2). This PI-II reaction centre in the tomato PI-II *ARPI* gene is CTKEC, but it became DAKEC in Pi7C, and WTKES in Pi6C. The second reaction centre in Pi6C is still intact, while in Pi7C it also mutated from the supposed CTKEC to CSKEC (the yellow region on the right blue head in Figure 2). In Pi6C, if the long chain from the N-terminal to the C3 area folded to the second reaction head, it would affect both function and specificity of the second reaction centre (Figure 2). Therefore, Pi6C has one centre knocked out and another centre could be affected, while Pi7C has one centre likely knocked out and one partially changed at the sequence level.

### Phylogenetic analysis of Pi6C and Pi7C and related PI-II peptides

**Figure 3:**
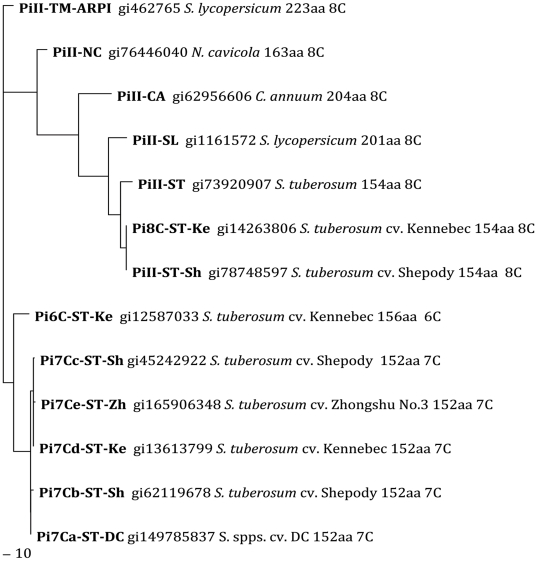
Protein Maximum Likelihood unrooted phylogenetic tree of PI-II genes and Pi7C and Pi6C natural variants. *Pi7Ca*-DC: *Pi7C* gene (EF469204) from the potato diploid clone 11379-03 that is essentially a *Solanum tuberosum* but with a background contribution from *S. phureja* and *S. stenotomum*. Other cultivars, Shepody, Kennebec, Zhongshu No. 3, are all tetraploid. Note that the Pi7C/Pi6C family cluster is separated from the standard 8C PI-II superfamily.

**Figure 4:**
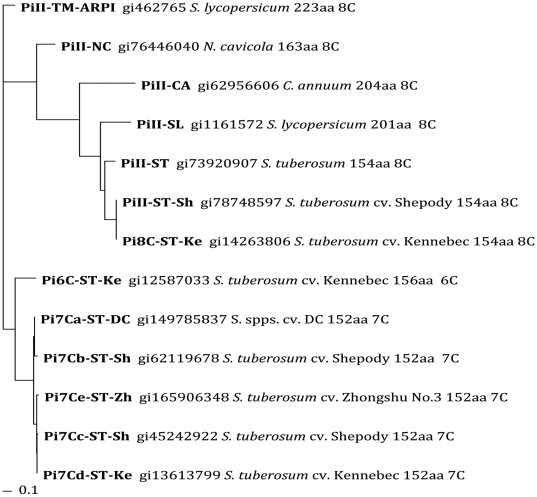
Protein NJ-1000 bootstraps phylogenetic tree (unrooted) of PI-II genes and Pi7C and Pi6C natural variants. Genotypes used are as the same in Figure 3. Note that the Pi7C/Pi6C family cluster is separated from the standard 8C Pi-II superfamily.

In the phylogenetic trees of the amino acid sequences, there were clearly two large clusters, in either the Maximum Likelihood tree or the Neighbour Joining tree (Figures 3, 4). One cluster was the standard PI-II members of 8C-PI-II proteins from tobacco (*N. cavicola*), pepper (*C. annuum*), tomato (formerly *Lycopersicum esculentum* now *Solanum lycopersicum* in GenBank), and potato (*S. tuberosum*). The other cluster, well separated as a new family, was all the natural variants with 7C or 6C domains, exclusively in potato (diploid and tetraploid) (Figures 3 and 4). When the tobacco (*N. cavicola*) was used as an outgroup, the 7C/6C cluster is close to ARPI-8C (Figure S1). The Pi6C and Pi7C forms two parallel groups, both related to the tomato ARPI on the phylogenetic tree, suggesting that *Pi6C* gene is likely not allelic to *Pi7C* gene in this cultivar Kennebec. Since none of the potato cDNA sequences with relatively high similarities to tomato *ARPI* can be decoded to a 8C-domain or to three PI-II domains either in our laboratory or GenBank, *ARPI* may not have a counterpart in the potato genotypes analyzed, or the counterpart is no longer expressed, or has been replaced by *Pi6C* or *Pi7C*.

### Ka/Ks value analysis

When *ARPI*, *Pi6C*, and *Pi7Ca* nucleotide sequences were analyzed for Ka/Ks ratios, a positive selection (Ka/Ks = 2.3937) was found for the evolution from the ancestor of *ARPI* and *Pi6C* to *Pi6C*. There was a mild negative selection (Ka/Ks = 0.6062) from the ancestor of *ARPI* and *Pi6C* to *ARPI*. The evolution from the ancestor of the three genes to Pi7Ca was also under positive selection (Ka/Ks = 1.5215). When the nucleotide sequences of the coding regions of all the proteins in this study are used, a positive selection from the ancestor to Pi6C was 1.6216 with a moderate negative selection (Ka/Ks = 0.6399) for the tomato ARPI (See Node 6 in Table S1 and Figure S2). Strong positive selections have been detected for Pi7Ce (Ka/Ks = 5.9259) and Pi7Cc (Ka/Ks = 2.5474) (See Node 8 Branch 2 and Node 9 Branch 1, respectively, in Table S1 and Figure S2).

## Discussion

### Natural PI-II variants with a loss of cysteine residues at the first reaction centre

It was formerly believed that there were no natural variants for the cysteine residues at either of the two reaction centres on PI-II, likely because of the essential role of the disulphide bonds for reaction centre structure [15]. In this study, the recently available expressed gene sequences allow us to identify five variants with 7 cysteine residues and one variant with 6 cysteine residues in the domain. Each of these natural variants had a full open reading frame, conserved N-end and C-end regions, and was expressed in the potato plants. These natural variants with only 7C or 6C in each domain form a new PI-II family within the PI-II superfamily. 

### Selective loss of cysteine residues and disulphide bonds and functionality of intermediate versions of the genes

In all six 7C/6C natural variants, the missing cysteine residues and disulphide bonds were all at the first reaction centre (Figure 2). Also, the lost cysteine residues were always in pairs, despite being distantly separately at the primary nucleotide sequence level in their genes (Figure 1). There were no remaining free cysteine residues in Pi7C and Pi6C protein members. This fact suggests that the loss of disulphide bonds is likely selective, not random, because both missing bonds are at the first reaction centre, although it is unknown why the second reaction centre was more conserved than the first centre during evolution. The results favour the possibility that the second reaction centre plays a more housekeeping role than the first reaction centre does for the PI-II protein. It is unclear whether the loss of the first cysteine residues for the missing bonds was random or selective at this stage, but the loss of the second cysteine residues was clearly not random as they were the mates of the first ones. It also indicates that intermediate versions of the genes or proteins were affected by selection pressure; not a neutral process.

### Evolutionary order of 6C domain and 7C domain compared to 8C domain

Tuber-bearing *Solanum* species are derived from non-tuberous species [25]. In the present study, Pi7C and Pi6C domains are identified exclusively in potato. The PI-II domains in other analyzed solanaceous plants are all of a typical Pi8C-type. Therefore, it is plausible to conclude that the Pi7C and Pi6C domains are derived from these typical 8C domains. The Pi7C members within cultivated diploid and tetraploid potatoes also suggest that the novel 7C domain is still differentiating to produce different alleles; likely partially influenced by agricultural selection in cultivated potatoes. The Pi7C and Pi6C domains are specific to the *S. tuberosum* potato, according to the information we have to date. Further study is required to investigate whether these domains in PI-II proteins exist in other *Solanum* species. If the *Pi7C* or *Pi6C* domain/genes can be found only in tuberous potato species, it would be interesting to determine whether there was a causal relationship, or simply a co-occurrence, between the loss of a pair of cysteines and the development of stem tubers.

### *Pi6C* and *Pi7C*: Functional genes or pseudogenes

The *Pi7C* gene is probably functional; all four *Pi7C* alleles have a full length open reading frame, active expression, and greater conservation within the domain regions than within the inter-domain regions. Similarly, the *Pi6C* gene is probably also functional with its full open reading frame and active expression. Although the transgenic approach sometimes cannot clarify the function of the gene tested, this approach has been used to confirm proteinase inhibitor functions, including potato plant resistance to insects [26] or detection of their role in seed development [27]. The proteins of Pi7C and Pi6C are cell wall-embedded and complicated our in vitro expression essay. Therefore, transgenic knockout or down-regulation using RNA interference technology or antisense technology might confirm the function of *Pi6C* and *Pi7C* in potato plants.

### Survival and differentiation of novel members in a gene family

The distribution of novel members of a gene family in populations is strongly influenced by selection pressure. In Brassica species, the male fertility-restorer genes for two male sterility systems (*nap* and *pol*) are alleles or haplotypes of the same locus. Which restorer gene is more frequently present in a given species or population is a reflection of their need to restore cytoplasmic male sterility in the species or population [28]. In the present study, the novel potato *Pi6C* was found in one of five potato genotypes while *Pi7C* occurred in all five. The parental intermediate versions for *Pi6C* and *Pi7C* were not found in these genotypes, likely due to selective loss because they had weaker beneficial response to selection than the *Pi6C* and *Pi7C*.

### *Pi6C* and *Pi7C* formation

The loss of paired cysteine residues located quite distantly at the primary sequence level suggests that the mutations did not occur in a single step. These cysteine residues are in pairs at the protein level (Figure 2), but gene mutation must be at the DNA level where the corresponding regions of the two cysteine residues are quite far apart (Figure 1). The DNA sequences of these two regions are quite different, suggesting that they were not changed simply by segment duplication or deletion. After one cysteine residue was lost, the unpaired cysteine residue would have caused paring issues for cysteine residues in other regions. For this reason it is very likely that the protein could not function well with an unpaired cysteine residue in PI-II. Relative stability of the protein would have been regained only when the counterpart cysteine residue of the pair was also lost. This process is likely to be a step-by-step adjustment in response to selection pressure during evolution. It is known that pseudo genes usually do not react to selection and will likely rapidly accumulate mutations, except occasionally some segments may be picked up into functional genes through recombination [29]. Most of the successive versions of the gene during the generation of *Pi6C* or *Pi7C* must have had somewhat beneficial functions for the plants during this mutation process in order for selection to occur and to avoid disruption of the open reading frames. In other words, they were rarely pseudo genes. The ratio of non-synonymous substitutions (Ka) to the rate of synonymous substitutions (Ks) can be used as an indicator of selective pressure acting on a protein-coding gene [30], [31]. A Ka/Ks ratio greater than 1.0 is usually indicative of positive selection pressure. The evolution from the ancestor to Pi6C and Pi7C clearly occurred under positive selection with Ka/Ks ratios (1.5215–2.3937) much greater than 1.0 (See Results). As the emergence of Pi7C and Pi6C genes was clearly under positive selection, their intermediate gene versions are very likely to have been functional. The functional-to-functional evolution inferred from the analysis of these novel genes in the PI-II gene family may provide insights into the evolutionary process of many other genes.

## Materials and Methods

### Plant materials

Potato diploid clone 11379-03 (DC) (essentially a *S. tuberosum* L.) [32] was used in this study. DC was derived from a number of crosses between different subspecies of the *S. tuberosum* group (*S. stenotomum*, *S. phureja*, and *S. tuberosum* ssp. *tuberosum*), and was developed at the Potato Research Centre, Agriculture and Agri-Food Canada (AAFC), Fredericton. Plant samples used for RNA isolation were collected from field-grown DC plants.

### Potato cDNA library construction and DNA sequencing

Plants of the DC used in cDNA library construction, were grown at the Benton Breeding Station of the Potato Research Centre. RNA was extracted according to Li et al. [33]. A cDNA library, LIPTC, was constructed with the lambda Uni-ZAP XR vector–Bluescript SK phagemid system and protocol with insertion sites *Eco*RI and *XhoI* (Stratagene, Vancouver, Canada) using mRNA of the main mass (storage parenchyma-perimedulla excluding the central pith) of immature tubers 85 days after planting. Random cDNA phagemid clones were sequenced from the 5′end, and some were confirmed with 3′end using the Applied Biosystems (ABI) BigDye Ready Reaction (Cat. # 4303152, California, USA) and an ABI DNA sequencer system. Assignment of putative function of the ESTs/genes was based on their homology with known genes in a BLAST search using the most recent releases of GenBank. For eliminating the errors associated with single-run EST sequencing, only the polymorphisms confirmed by at least one other EST were used for detailed analysis.

### Identification of the putative PI-II cDNA and bioinformatic analysis

A cDNA clone with serial number C463 (the allele later called *Pi7Ca* in this article) in our laboratory in AAFC was identified as a putative PI-II and then re-sequenced in both directions with an ABI DNA sequencer. Nucleotide sequences were translated into amino acid sequences using http://insilico.ehu.es/translate [34]. The protein sequence was used in “position-specific iterated and pattern-hit initiated BLAST” (PSI- and PHI-BLAST) [35], [36] to identify similar proteins and conserved known domains, which were then aligned to show the existence or absence of cysteine residues. The File S1 presents the tabulated list of proteins (or the source mRNA IDs for the translated amino acid sequences) that were used in this alignment. The domain's known structure was viewed with Cn3D 4.1 (an NCBI's helper application, http://www.ncbi.nlm.nih.gov/Structure/CN3D/cn3d.shtml). The amino acid alignment between PI-II members was exported to a FASTA file and further treated with BiEdit Sequence Alignment Editor (version 7.0.5.3) [37] (http://www.mbio.ncsu.edu/bioedit/bioedit.html).

### Phylogeny analysis

Protein sequences were used in phylogenetic analysis. The protein sequences were downloaded from NCBI (http://www.ncbi.nlm.nih.gov) if they had an available ID in GenBank. For expressed sequences without available GenBank ID, the cDNA sequences were translated into protein sequences using the insilico.ehu.es website (http://insilico.ehu.es/translate) [34]. In addition to the known PI-II proteins with 8-cysteines in each domain in potato (*S. tuberosum*) and its close relatives tomato (*S. lycopersicum*) and pepper (*C. annuum*), all the abnormal PI-II sequences were used in phylogenetic analysis as long as their amino acid sequences showed at least one cysteine residue missing. The tobacco (*N. cavicola*) was used as an outgroup in one of the trees because it has sufficient similarity with both Pi7C and PI-II genes to allow the phylogenetic tree to stay within a reasonable size. Their amino acid sequence phylogenetic trees were reconstructed using BioEdit [37] using amino acid sequences (File S2). Two approaches were used in reconstructing the phylogenetic tree: One was Clustal [38]-ProML option—conducting the ClustalW Multiple Alignment (File S3) and then reconstructing the Protein Maximum Likelihood tree (File S4). The other approach was to use the “ClustalW Multiple Alignment” and 1000 “bootstraps NJ tree” option to do alignment and then generate the phylogenetic tree (File S5) with Protdist Neighbor Phylogenetic Tree Option (PROTDIST version 3.5c) (an option in BioEdit). The outtree files were saved as .txt files and then opened with TreeView (win32) 1.6.6. (http://taxonomy.zoology.gla.ac.uk/rod/rod.html) and then saved as graphics.

### Ka/Ks value analysis

The ratios of the rates of non-synonymous substitutions (Ka) to the rates of synonymous substitutions (Ks), as described by Liberles [30], [31] were calculated using the Ka/Ks Calculator Tool http://services.cbu.uib.no/tools/kaks. The input nucleotide sequences were the mRNA region encoding the proteins used in phylogenetic tree analysis (File S6). The Ka/Ks Calculator Tool calculated the multiple sequence alignment and the phylogenetic tree from these input sequences, translated the DNA-sequences to protein, calculated the alignment, transformed it back to DNA, and aligned it on codon boundaries for Ka/Ks calculation. This alignment was used to calculate a phylogenetic tree using a least squares distance method with Jukes and Cantors distances on the DNA. For additional detail, the reader is referred to http://services.cbu.uib.no/tools/kaks/docs/kaksfields#phyl0.

## Supporting Information

Figure S1 **Protein NJ-1000 bootstraps phylogenetic tree, rooted using the tobacco PiII8C as the outgroup, of PI-II genes and Pi7C and Pi6C natural variants.** Genotypes used are the same in Figure 3. Note that the tomato ARPI is close to the Pi7C/Pi6C family.
(TIF)

Figure S2 **A phylogenetic tree of mRNA sequences that encode PI-II and Pi7C/6C proteins, reconstructed by the Ka/Ks Calculator Tool (** http://services.cbu.uib.no/tools/kaks **).**
(TIF)

Table S1 **Ka/Ks values for each node of the tree in Figure S2.**
(DOC)

File S1 **The IDs and plant species of proteins or the source mRNA sequences used in** **Figure 1** **.**
(TXT)

File S2 **The input amino acid sequences for phylogenetic tree reconstruction.**
(TXT)

File S3 **The ClustalW Multiple Alignment file of amino acid sequences used in phylogenetic tree reconstruction.**
(DOC)

File S4 **The tree file for** **Figure 3** **“Protein Maximum Likelihood unrooted phylogenetic tree of PI-II genes and Pi7C and Pi6C natural variants.”**
(DOC)

File S5 **The tree file for** **Figure 4** **“ Protein NJ-1000 bootstraps phylogenetic tree (unrooted) of PI-II genes and Pi7C and Pi6C natural variants.”**
(DOC)

File S6 **The input file of the mRNA coding sequences for Ka/Ks analysis.**
(TXT)
