# Supplementary figures and images for: Selective Loss of Cysteine Residues and Disulphide Bonds in a Potato Proteinase Inhibitor II Family
Source: PLoS One. 2011 Apr 11;6(4):e18615. doi: 10.1371/journal.pone.0018615 (PMC3073943; doi:10.1371/journal.pone.0018615)

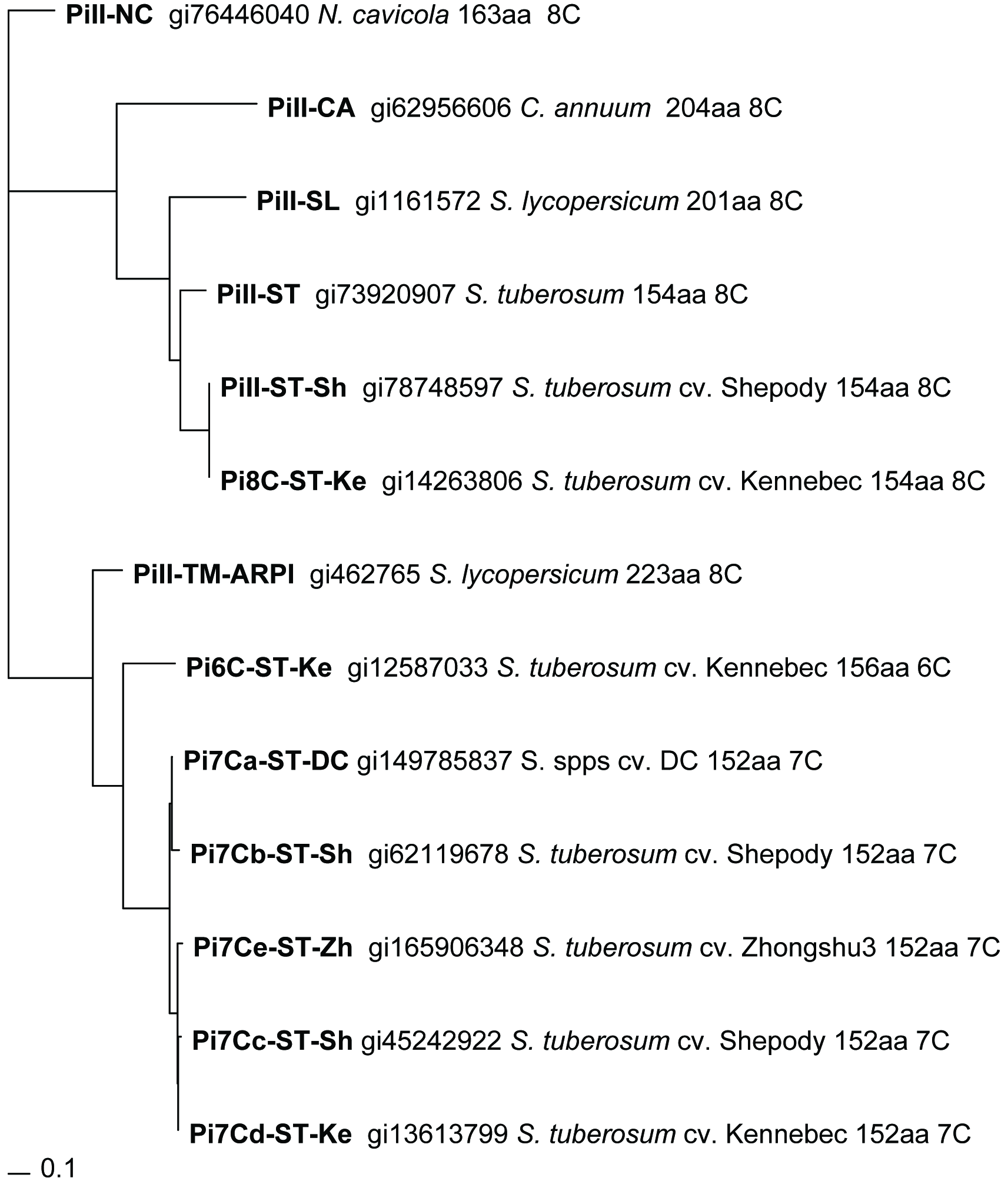

Supplement: Figure S1 — Protein NJ-1000 bootstraps phylogenetic tree, rooted using the tobacco PiII8C as the outgroup, of PI-II genes and Pi7C and Pi6C natural variants. Genotypes used are the same in Figure 3. Note that the tomato ARPI is close to the Pi7C/Pi6C family. (TIF) [file pone.0018615.s001.tif]

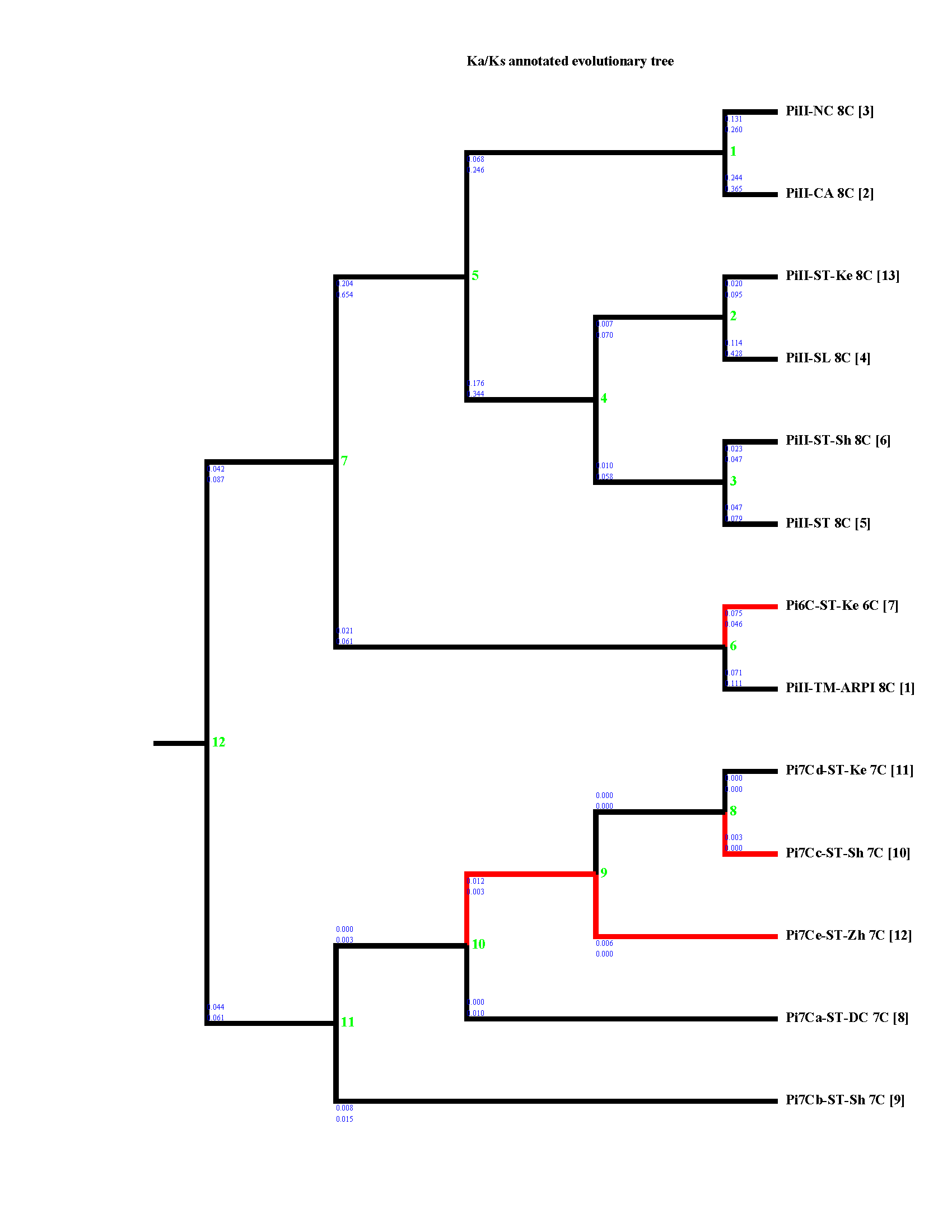

Supplement: Figure S2 — A phylogenetic tree of mRNA sequences that encode PI-II and Pi7C/6C proteins, reconstructed by the Ka/Ks Calculator Tool ( http://services.cbu.uib.no/tools/kaks ). (TIF) [file pone.0018615.s002.tif]
