# Supplementary material for: Selective Loss of Cysteine Residues and Disulphide Bonds in a Potato Proteinase Inhibitor II Family
Source: PLoS One. 2011 Apr 11;6(4):e18615. doi: 10.1371/journal.pone.0018615 (PMC3073943; doi:10.1371/journal.pone.0018615)
Supplement: Table S1 — Ka/Ks values for each node of the tree in Figure S2. (DOC) [file pone.0018615.s003.doc]

**Table S1**. Ka/Ks values for each node of the tree in Figure S2

| Node | Ka/Ks Branch1 | Ka Branch1 | Ks Branch1 | Ka/Ks Branch2 | Ka Branch2 | Ks Branch2 |
| --- | --- | --- | --- | --- | --- | --- |
| 1 | 0.5016 | 0.1306 | 0.2605 | 0.6696 | 0.2444 | 0.365 |
| 2 | 0.2153 | 0.0205 | 0.0951 | 0.2672 | 0.1145 | 0.4284 |
| 3 | 0.4825 | 0.0226 | 0.0469 | 0.5999 | 0.0474 | 0.0789 |
| 4 | 0.1033 | 0.0073 | 0.0705 | 0.1713 | 0.0100 | 0.0582 |
| 5 | 0.2777 | 0.0683 | 0.2461 | 0.5127 | 0.1763 | 0.3438 |
| 6 | 1.6216 | 0.0745 | 0.0460 | 0.6399 | 0.0707 | 0.1105 |
| 7 | 0.3117 | 0.2039 | 0.6542 | 0.3423 | 0.02078 | 0.0607 |
| 8 | 0.0000 | 0.0000 | 0.0000 | 2.5474 | 0.0026 | 0.0000 |
| 9 | 5.9259 | 0.0059 | 0.0000 | 0.0000 | 0.0000 | 0.0000 |
| 10 | 3.7212 | 0.01191 | 0.0032 | 0.0000 | 0.0000 | 0.0097 |
| 11 | 0.5784 | 0.0085 | 0.0147 | 0.0000 | 0.0000 | 0.0032 |
| 12 | 0.4821 | 0.0420 | 0.0871 | 0.7177 | 0.0439 | 0.0612 |
